# Risk factors for intra-abdominal hypertension and abdominal compartment syndrome in patients admitted to the ICU

**DOI:** 10.1186/cc10214

**Published:** 2011-06-22

**Authors:** M Assunção, FS Oliveira, BF Mazza, F Freitas, M Jackiu, FR Machado

**Affiliations:** 1 Escola Paulista de Medicina, Federal University of São Paulo, UNIFESP, São Paulo - SP, Brazil

## Introduction

Intra-abdominal hypertension (IAH) and abdominal compartment syndrome (ACS) as well as their risk factors were defined recently by consensus. These diseases have a high incidence and morbi-mortality in patients admitted to the ICU and represent a huge problem among critically ill patients.

## Objective

To determine the incidence of IAH or ACS in patients admitted to a university hospital ICU with two or more risk factors.

## Methods

All patients admitted to the ICU were evaluated daily. Those with at least two risk factors were submitted to intraabdominal pressure (IAP) monitoring by intravesical pressure method once daily, during 7 days or until death or ICU discharge. In each measure, the abdominal perfusion pressure (APP) (that is, IAP - mean arterial pressure) was recorded. Demographic data, APACHE II, ICU and hospital length of stay and mortality were determined. Results are presented as the percentage or mean ± standard deviation (Table 1).

**Table 1:** Characteristics of the study participants and mortality

Age	66 (± 17)
Female gender, % (*n*)	34 (11)
APACHE II score, mean (±SD)	18 (± 4)
Emergency surgery, % (*n*)	0 (13)
Elective surgery, % (*n*)	22 (7)
Medical, % (*n*)	38 (12)
IAH, % (*n*)	62 (20)
ACS, % (*n*)	12.5 (4)
Length of stay (days)	
Hospital	60 (± 55)
ICU	14 (± 15)
Mortality (%)	
28-day	31
ICU	38
Hospital	62

## Results

Patients were assessed from February 2010 to October 2010 and 164 were enrolled. Thirty-two patients fulfilled criteria for IAP monitoring (mean age 62 ± 17 years, 37% (12) female, mean APACHE II score 18 ± 4). Among these 32 patients, 62% (20) had at least one IAH episode and 12.5% (four) developed ACS. Only patients with ACS had APP <60 mmHg. Hospital LOS was 60 ± 55 days, ICU LOS was 14 ± 15 days. The 28-day, ICU and hospital mortalities were 31% (10), 38% (20) and 62% (20), respectively.

## Conclusion

Risk factors have a high incidence in our ICU. IAH/ACS patients present a high mortality and a long LOS.

